# Bilateral Ovarian Torsion during Follow-up for Antenatally Detected Ovarian Cysts

**DOI:** 10.21699/ajcr.v8i3.563

**Published:** 2017-05-01

**Authors:** Bozidar Zupancic, Marko Baskovic, Ljudevit Sovic, Dubravko Habek

**Affiliations:** 1 Department of Pediatric Surgery, Children's Hospital Zagreb, Croatia; 2 Department of Gynecology and Obstetrics, University Hospital Sveti Duh, Croatia

**Keywords:** Ovarian cyst, Antenatal diagnosis, Ovarian torsion

## Abstract

Ovarian torsion is a surgical emergency demanding timely diagnosis and treatment to prevent loss of the ovaries which if happens may result in functional and emotional consequences. Simple (less than 5cm in size) ovarian cysts require follow-up for potential self-resolution. We describe a case of antenatally detected bilateral ovarian cysts that developed bilateral ovarian torsions on follow-up, postnatally.

## INTRODUCTION

Antenatally detected ovarian cysts can be classified into simple and complex cysts. Simple ovarian cysts with size less than 5cm can resolve itself over few weeks to months postnatally, owing to decrease in levels of maternal hormones in patient’s blood. Complex cysts or ovarian cysts with size more than 5cm are usually dealt surgically to avoid gonadal loss.[1] We report a patient in whom drastic complication occurred while patient was under follow up.

## CASE REPORT

A female newborn, product of a twin pregnancy (36.5 weeks of gestation), was discharged on10th day after the birth for follow-up due on antenatally detected bilateral ovarian cysts (size less than 5cm). On follow-up at the age of 2 months, pelvic ultrasound showed right ovary 37x27x35 mm in size and left ovary 29x32x50 mm size filled with echogenic content with intraluminal septa and normal circulation on Doppler ultrasound. Tumor markers done were reported as ß-HCG 1.4 IU/L and alpha fetoprotein 732.9 ng/mL. Patient was advised for regular follow-up. Due to vague unrest of infant and suspected signs of acute abdominal pain, twenty days after discharge, the infant was readmitted. Ultrasound findings suggested bilateral adnexal torsion without detectible blood flow on Doppler ultrasound. Emergent exploration showed the left ovary of approximately 5 cm in diameter, twisted by 720 degrees and the right ovary twisted around its axis by 720 degrees both were of dark brown in color. The right ovary with its stem was wrapped around the left fallopian tube (Fig.1). Initially, detorsion of both ovaries was done. After half an hour of waiting and applying warm packs, ovaries appeared gangrenous, except for a small segment of left ovary. Considering the intraoperative findings right-sided adnexectomy and left-sided partial oophorectomy was done leaving 0.5 cm2 of ovarian tissue behind. Histopathology findings reported cysts with tissue structure barely discernible due to coagulation necrosis. On third postoperative day, re-laparotomy and intestinal adhesiolysis was done owing to early postoperative intestinal obstruction. The residual ovarian tissue looked normal. Postoperative course was uneventful this time. Three years after surgery, ultrasound of residual ovarian tissue shows a typical echo-structure of the ovary 12x10 mm with detectable flow in adnexal pedicle.

**Figure:**
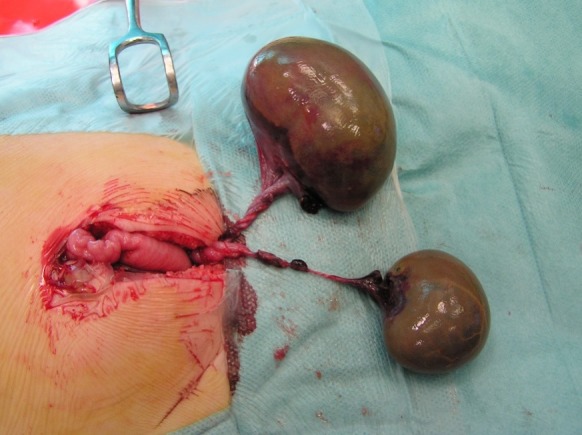
Figure 1: Bilateral ovarian torsion with adnexal entanglement.

## DISCUSSION

Few reports described ovarian torsion during intrauterine period with simple cysts of size less than 5cm. Our patient was in follow up for self resolution of ovarian cyst however, she developed torsion. If symptoms were picked up early, a prompt surgical intervention would have saved the ovaries, though a small portion of left ovary remained intact and is growing on follow-up scans.

In our case, both ovaries not only had multiple twists around their axes, but also these ovaries were wrapped around each other. Only few cases of ovarian entanglement have been reported in literature.[2-5] Enlarged cysts with the elongation of both adnexal ligaments may be the possible explanation for the torsion and entanglement. Possibility of torsion in small sized simple ovarian cyst must be kept in mind in patients who are on follow up for spontaneous resolution and same must be told to the parents.

## Footnotes

**Source of Support:** Nil

**Conflict of Interest:** None declared

